# The relationship between depression and risk of metabolic syndrome: a meta‐analysis of observational studies

**DOI:** 10.1186/s40842-021-00117-8

**Published:** 2021-03-02

**Authors:** Yousef Moradi, Ahmed N Albatineh, Hassan Mahmoodi, Reza Ghanei Gheshlagh

**Affiliations:** 1 Social Determinants of Health Research Center, Research Institute for Health Development, Kurdistan University of Medical Sciences, Sanandaj, Iran; 2 Department of Community Medicine and Behavioral Sciences, Faculty of Medicine, Kuwait University, Kuwait City, Kuwait; 3 Spiritual Health Research Center, Research Institute for Health Development, Kurdistan University of Medical Sciences, Sanandaj, Iran

**Keywords:** Depression, Metabolic syndrome, Observational study, Meta‐analysis

## Abstract

**Introduction:**

The link between metabolic syndrome and depression has always been controversial. Different studies that have examined the relationship between metabolic syndrome and depression have reported different results. Therefore, the goal of the present study was to examine the association between depression and MetS by meta-analysis.

**Methods:**

Embase, Scopus, PubMed, and ISI were searched for publications in English from January 1990 to February 2020. Search included cohort and cross-sectional studies aimed at examining the association between depression and MetS. The risk of bias was assessed by Newcastle-Ottawa Scale. Heterogeneity and publication bias were tested, subgroup analysis and meta-regression were conducted.

**Results:**

49 studies with total sample size 399,494 were analyzed. Results indicated the odds of MetS was higher in depressed compared to non-depressed individuals [OR: 1.48; 95 %CI: 1.33–1.64) vs. (OR: 1.38; 95 %CI: 1.17–1.64)]. For cross-sectional studies, depressed patients in Europe (OR = 1.35; 95 %CI: 1.47–1.99) were at higher odds of MetS compared to those in America and Asia. For cohort studies, depressed patients in America (OR = 1.46; 95 %CI: 1.16–1.84) were at higher odds of MetS than those in Europe. Cross-sectional studies indicated women with depression were at higher odds of MetS (OR = 1.95; 95 %CI: 1.38–2.74) compared to men. In both types of studies, the odds of MetS decreased with age.

**Conclusions:**

Metabolic syndrome is more common in depressed compared to non-depressed individuals.

## Introduction

Metabolic syndrome (MetS) is a cluster of conditions, such as increase in waist circumference, dyslipidemia (elevated triglyceride levels and reduced HDL), increased blood pressure, and increased fasting blood sugar levels that is related to insulin resistance, diabetes, and elevated risk of cardiovascular disease [1, 2]. MetS and related metabolic biomarkers may be related to mood disorders, including depression [3].

Sedentary lifestyle and poor diet are known as the main causes of MetS that may be more common in depressed individuals compared to non-depressed people [4]. The high co-occurrence between MetS and mood disorders indicates high pathophysiological overlap between the two conditions [3]. The global prevalence of depression is increasing, and it is projected to become the second-leading cause of death by 2030 [5, 6].

There are hypothetical mechanisms by which there may be a link between MetS and depression. Due to low physical activity, patients with depression are vulnerable to weight gain, MetS, and finally diabetes and cardiovascular diseases [7]. Previous studies on the association between MetS and depression have led to conflicting results. Some studies have found no significant association between the two conditions [8–12]; while others have found significant associations [13]. In light of the existing controversies about the relationship between these two variables, we felt that the literature on this subject needed to be re-evaluated. Therefore, the present systematic review and meta-analysis aims to provide pooled estimate of the association between MetS and depression which will offer an evidence-based answer to the association between depression and MetS can guide clinical management and therapy in decision making [14].

## Methods

The present systematic review and meta-analysis was conducted according to the Preferred Reporting Items for Systematic Reviews and Meta-Analyses (PRISMA) according to the following 5 steps: Search Strategy and Search Terms, Eligibility Criteria and Study selection, Data Extraction, Quality Assessment and Risk of Bias, and Meta- analysis [15].

### Search strategy and search terms

All internal databases, such as PsycInfo, Cochrane, Web of Sciences, Scopus, and Pub Med (Medline) were searched for articles published from January 1990 to February 2020. Grey literature (conference papers, related magazines etc.) was also examined. The search was conducted using the following keywords that were selected using Mesh and Emtree:

“Syndrome X”, “Metabolic Syndrome”, “Insulin Resistance Syndrome”, “MetSyn”, “Depression”, “Depressive Disorder”, “Dysthymic disorder”. The search process was conducted by two independent researchers (RGH and YM); any disagreement between them was resolved through discussion.

### Eligibility criteria and study selection

At the end of the search process, all selected articles were included in EndNote, version 8. First, duplicate articles were identified then screened based on titles and abstracts. Second, full texts were examined, and the final articles were selected. The inclusion criteria were as follows: original observational cohort or cross-sectional studies, focused on depression as exposure, risk of MetS as outcome, and published in English. Case or case-report studies, letters to the editor, series, randomized clinical trials, studies not reporting the risk of MetS in depressed patients using the indices of effect size, such as odds ratio and risk ratio, articles without available fill texts, and articles published in languages other than English were excluded.

### Data extraction

In the first step, titles and abstracts and in the next step, full texts were reviewed to extract the required information, such as name of the first author, year and country of publication, depression screening tools, diagnostic criteria of MetS, mean age of participants, number of patients with depression, number of patients with MetS. List of references for articles were also examined to identify other potential articles.

### Quality assessment and risk of bias

The methodological quality of articles was examined by two independent researchers (RGH and YM) based on the 10 items in STROBE checklist. The 10 items assessed: title and abstract, objectives and hypotheses, inclusion criteria, sample size, statistical methods, descriptive data, interpretation of results, study limitations, and funding [16]. Higher scores on this checklist indicated better methodological quality. According to this score, articles were divided into three categories of methodological quality, including poor (4 or below), average [4–7] and good (over 7). Risk of bias was assessed using the Newcastle-Ottawa Quality Assessment Scale (NOS) [17]. The NOS assesses any study using 6 items in 3 groups, including selection, exposure, and comparability. The maximum score on the NOS is 9. When there are differences in the scores given to the selected articles, this is resolved through external discussion.

### Statistical analysis

In the cross-sectional studies, the effect size of depression on MetS was reported using qualitative dichotomous (Yes-No) scores together with the odds ratio (OR). Logarithm and standard error of OR were used to combine the results of studies. ORs were combined with standard error of ORs using the random effects model. In cohort studies, the effect size of depression on MetS was reported using qualitative dichotomous (Yes-No) scores together with the risk ratio (RR); RRs were combined with standard error of RRs using the random effects model. For studies in which results were reported as percentages, first, the variance and standard error of each study were calculated using the binomial distribution, and then, the random or fixed effects model was used to combine the results of different studies. Heterogeneity among the studies was examined using the Cochran’s Q test and the DerSimonian and Laird’s statistical method. Cochran Q and I square (I^2^) tests were used to investigate the heterogeneity and variance between studies. The Q statistic tells us whether there is statistically significant heterogeneity among the studies. The I^2^ value indicates the amount of heterogeneity quantitatively in a range from 0–100 %. Thresholds for the interpretation of I^2^ can be misleading, since the importance of inconsistency depends on several factors. A rough guide to interpretation is 0–40 % (might not be important), 30–60 % (may represent moderate heterogeneity), 50–90 % (may represent substantial heterogeneity), and 75–100 % (considerable heterogeneity) [18]. In case of heterogeneity among studies, the random effects model was used to combine the results [19]. A funnel diagram, Egger’s test, and its graphs were used to evaluate the publication bias. In the Egger’s regression model, the ratio of the effect size on the standard error, which is the standard index (z-score), is taken as the dependent variable and predicts its value over the inverse of standard error (1/SE) [19]. Forest plot was produced, and meta-regression was conducted to examine the association between sample size, year of publication, and mean age of participants with odds of MetS. Additionally, subgroup analysis was conducted by gender (women, men, and both), depression screening tool, diagnostic criteria of MetS (WHO, IDF, ATP III), population (patients, general population), mean age of patients (below or over 50 years), and geographical location (Asia and Australia, Europe and America). Some studies reported their results by gender or instrument rather than reporting total prevalence rates; for these studies, two groups were included in the analysis. The data was analyzed using Stata, version 16.

## Results

**Fig. 1:**
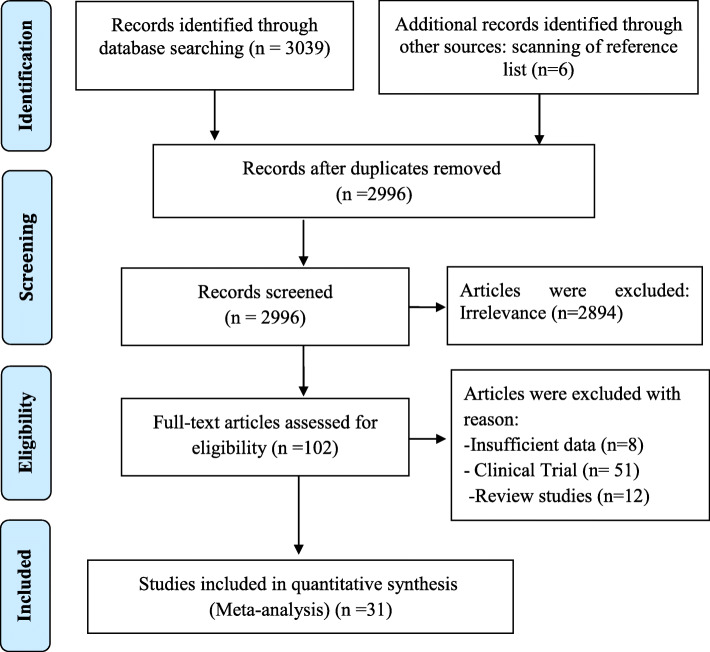
Process of searching for, screening, and selecting the eligible articles

In the initial search, 2996 non-duplicate articles were identified that were potentially eligible. In the screening process, 146 articles were maintained after excluding the unrelated ones. In the next step, the eligibility of articles was examined; of which 102 articles were excluded, and 31 articles were included in the final analysis. Table 1 shows the process of screening and selecting the articles. (Fig. 1)

### Study and participants’ characteristics

Overall, 49 articles (31 cross-sectional studies in 36 groups and 18 cohort studies in 21 groups) were included in the analysis. The total sample sizes of the cross-sectional and cohort studies were 111,866 and 287,628, respectively. Most of cross-sectional studies (4 studies in each country) had been conducted in Brazil [3, 20–22] and South Korea [23–26]; and most cohort studies (n = 6) had been conducted in America [7, 27–31].

Among cross-sectional studies, the highest and lowest sample sizes were for the studies by Park [26] (*n* = 23,385) and Chattopadhyay [32] (*n* = 97), respectively; and in four studies, the sample included over 10,000 participants [11, 24, 26, 33]. Among cohort studies, the highest and lowest sample sizes were for the studies by Dregan [34] (*n* = 124,445) and Viinamaki [35] (*n* = 223), respectively. Results were reported by gender in four studies [13, 25, 36, 37] and by type of depression (typical vs. atypical) in one study [38]; these studies were included in the analysis as two separate studies. In addition, three cross-sectional studies reported their results by gender; these were included in the analysis as three separate studies [7, 34, 39]. One cross-sectional study was focused on nursing personnel [22], and one on office workers [1]. Participants’ type of illness was clearly stated only in two studies, one among patients with mental disorders [40] and one among patients with type II diabetes [41]. Among 21 cohort studies, 19 were among the general population, one was among patients with cardiovascular disease [31], and one was among office staff [42]. Further details are provided in Table 1.

**Table 1:** Main characteristics of the selected studies

Study Type	First Author	Year	Sample size	Age	Country	Target	Scale		NOS Score
							Depression	MetS	
Cross-sectional studies	Ko [23]	2019	9867	≥ 19	Korea	General population	PHQ-9	ATP III	8
	Moreira [20]	2019	545	18–24	Brazil	Young adults	Interview	ATP III	6
	Chattopadhyay [32]	2018	97	-	India	Primary care patients	BDI	ATP III	7
	Bakhtiari [43]	2018	1560	69.3 ± 7.4	Iran	Elderly people	GSD	ATP III	7
	Mattei [44]	2018	129	40–80	Italy	Primary care patients	HADS	ATP III	7
	Kim [24]	2018	10,459	-	Korea	General population	PHQ-9	ATP III	7
	Moreira [21]	2017	972	25.8 ± 2.1	Brazil	General population	Interview	ATP III	7
	Ra [25]	2017	1938	≥ 40	Korea	Men	Interview	ATP III	7
			2404			Women			7
	Yu [11]	2017	11,430	≥ 35	China	General population	PHQ-9	ATP III	8
	Chang [12]	2017	11,258	-	Taiwan	General population	MHI-5	WHO	8
	Cardenas [45]	2017	332	≥ 60	USA	Elderly	PHQ-9	WHO	8
	Park [26]	2016	23,385	46.13 ± 0.18	Korea	Women	Interview	ATP III	8
	Agarwal [40]	2016	150	-	India	psychiatric Outpatient	Interview	ATP III	7
	Ribeiro [22]	2015	226	23–66	Brazil	Nursing personnel	HADS	ATP III	7
	Kahl [46]	2015	163	-	Germany	Primary care patients	Interview	ATP III	7
	Vargas [3]	2014	342	-	Brazil	General population	Interview	IDF	7
	Butnoriene [47]	2014	1115	62 ± 9.6	Lithuania	General population	Interview	ATP III	7
	Takeuchi [38]	2013	1011	41.1 ± 8.1	Japan	General population	Interview	IDF	8
			1011	42.3 ± 8.7					8
	Sekita [13]	2013	1353	63	Japan	Men	CES-D	ATP III	7
		2013	1760	62		Women			7
	Marijnissen	2013	1277	61.1 ± 5.9	Netherlands	General population	BDI-I	IDF	7
	Demirci [48]	2011	250	-	Turkey	General population	BDI	ATP III	7
	Foley [8]	2010	2525	-	Australia	General population	Interview	ATP III	8
	Ahola [41]	2010	1226	45 ± 12	Finland	Diabetes type I	BDI	ATP III	8
	Hildrum [9]	2009	9571	47.74	Norway	General population	HADS	IDF	8
	Takeuchi [1]	2009	1215	42.5	Japan	Office workers	POMS	IDF	8
	Toker [36]	2008	1525	20–75	Israel	Women	PHQ-9	ATP III	7
			2355	20–75		Men			7
	Miettola [49]	2008	416	50.4 ± 10.5	Finland	General population	BDI	ATP III	6
	Dunbar [50]	2008	1345	25–84	Australia	General population	HADS	ATP III	7
	Vogelzangs [51]	2007	867	74.1 ± 6.6	Italy	General population	CES-D	ATP III	7
	Skilton [52]	2007	1598	51.8 ± 9.8	France	Primary care patients	HADS	ATP III	7
	Kinder [37]	2004	3003	28.7	USA	Women	Interview	ATP III	7
			3186	28.2		Men			7
Cohort studies	Dregan [34]	2020	71,799	-	United Kingdom	Women	Interview	ATP III	8
			52,646	-		Men			8
	Matta [4]	2019	64,861	46.30	France	General population	CES-D	IDF	7
	van Leijden [2]	2018	21,182	44.2 ± 13.2	Netherlands	Multi-ethnic	PHQ-9	IDF	7
	Rethorst [27]	2017	47,702	-	USA	General population	Interview	ATP III	7
	Marriana [53]	2017	1172	62	Finland	General population	CES-D	ATP III	7
	Matthew [28]	2016	1743	52.5	USA	General population	CES-D	ATP III	7
	Renel [29]	2015	1798	43.3	USA	General population	CES-D	ATP III	7
	Akbaraly [54]	2011	4446	≥ 65	France	Elderly	CES-D	ATP III	7
	East [7]	2010	1688	47.6 ± 10.3	USA	Women	CES-D	ATP III	7
			3437	49.3 ± 10.2		Men			7
	Akbaraly [42]	2009	5232	49.5 ± 6.1	France	Office staff	GHQ	ATP III	8
	Goldbacher [30]	2009	429	45.6	USA	Women	DSM-IV	ATP III	7
	Vogelzangs [10]	2009	1212	55–85	Netherlands	General population	CES-D	ATP III	8
	Vanhala [39]	2009	294	45.9	Finland	Women	BDI	ATP III	6
			194	46		Men			6
	Viinamaki [35]	2009	223	-	Finland	General population	DSM-IV	ATP III	6
	Vaccarino [31]	2008	652	-	USA	Cardiovascular disease	BDI	ATP III	7
	Katri [55]	2007	432	49	Finland	General population	BDI	ATP III	7
	Gil [56]	2006	795	-	Poland	General population	BDI	ATP III	7
	Herva [57]	2006	5691	-	Finland	General population	HSCL-25	ATP III	8

Abbreviations: *BDI-I* Beck Depression Inventory; *CES-D* Center for Epidemiologic Survey-Depression; *DSM* Diagnostic and Statistical Manual of Mental Disorders; *GHQ* General Health Questionnaire; *GSD* Geriatric Depression Scale Hopkins Symptom Checklist; *HSCL* Hopkins Symptom Checklist; *IDF* International Diabetes Federation; *MHI-5* 5-item Mental Health Inventory; *MINI* Mini International Neuropsychiatric Interview; *NCEP-ATP III* National Cholesterol Education Program-Adult Treatment Panel III; *PHQ* Patient Health Questionnaire; *POMS* Profile of mood states; *WHO* World Health Organization

### Meta‐analysis of cross‐sectional studies

#### Depression and risk of metabolic syndrome

**Fig. 2:**
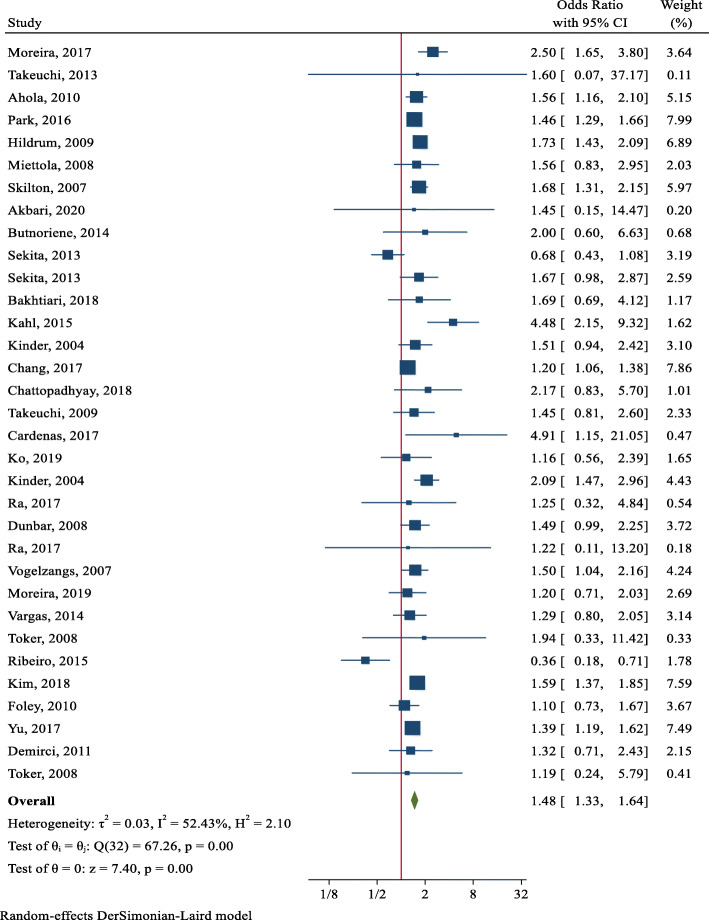
Forest plot for the pooled estimate of the Odds Ratio for the association between depression and MetS in the general population

Overall, the results showed that the pooled OR of MetS in patients with depression was 1.48 % (95 % CI: 1.33–1.64). Heterogeneity was found to be I^2^ = 52.43 %, and the Cochran’s Q test led to a statistically significant result (Q = 67.26, DoF = 32, *p* = 0.003) (Fig. 2).

**Fig. 3:**
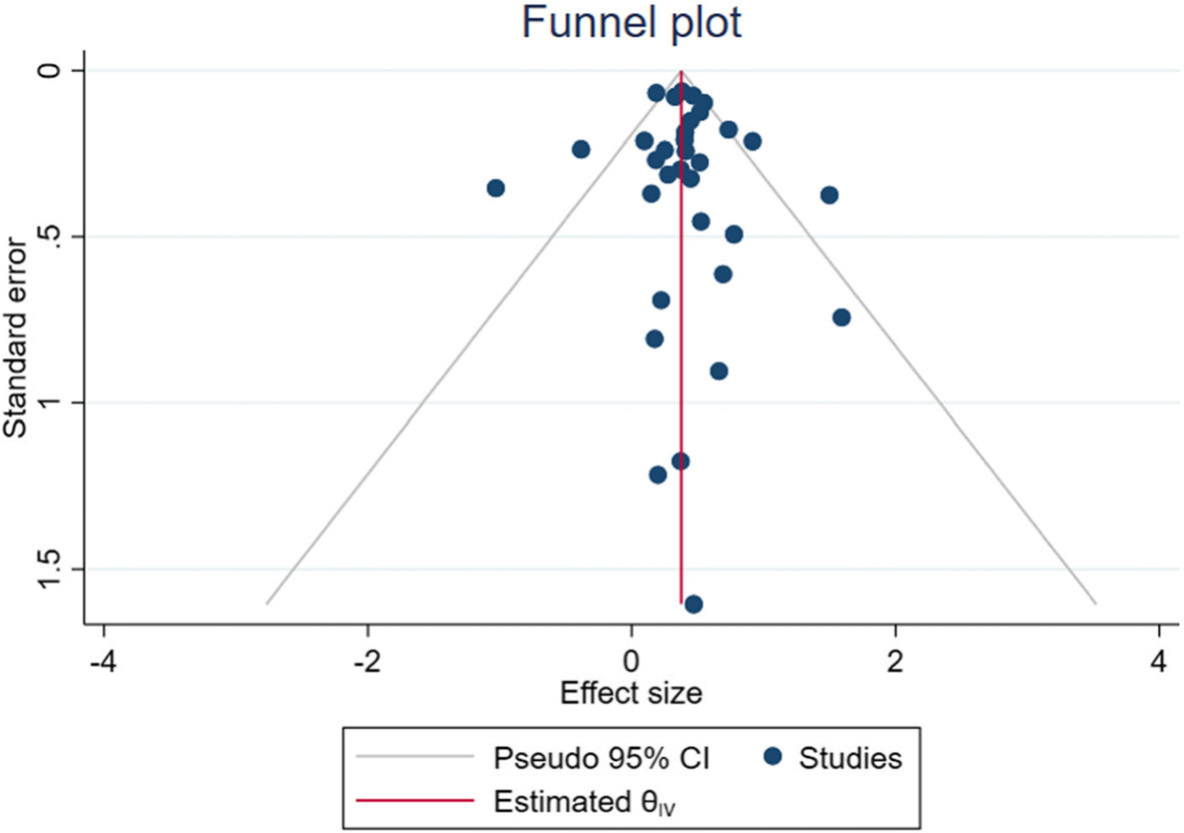
Funnel plot for testing publication bias in the pooled estimate of the association between depression and MetS in the general population

Also, the meta-regression indicated that the log odds of MetS in patients with depression significantly decreased with age (estimated β: -0.017, SE: 0.007, *p* = 0.021, 95 % CI -0.033, − 0.002). This means for every year increase in age, there is 1.7 % decrease in the odds of developing MetS in patients with depression (OR = 0.983, 95 % CI: 0.968, 0.998) and so older age has a protective effect against MetS in depressed patients. In addition, the funnel plot and the results of the Egger’s test (Coefficient: 0.09, SE: 0.394, *p* = 0.794) indicated no publication bias exists in the studies (*p* = 0.590) (Fig. 3).

#### Subgroup analysis

Results of subgroup analysis by geographical location showed that the pooled ORs of the studies conducted in Europe (OR = 1.71; 95 % CI: 1.47–1.99) were higher compared to the studies conducted in America (OR = 1.45; 95 % CI: 0.94–2.25) and Asia (OR = 1.37; 95 % CI: 1.26–1.49). Heterogeneity was only significant for studies conducted in America (I^2^ = 79.28 %, *p* = 0.010). Results by gender indicated that depressed men were more likely to develop MetS compared to depressed women [(OR = 1.52; 95 % CI: 1.13–2.05) vs. (OR = 1.35; 95 % CI: 0.90–2.01)], though women pooled estimate is not significant. In addition, the results of subgroup analysis showed that pooled ORs were higher in the studies that used interview to examine depression (OR = 1.70; 95 % CI: 1.37–2.12) (compared to questionnaires), and that the pooled ORs of the WHO’s diagnostic criteria of MetS (OR = 2.0; 95 % CI: 0.53–7.52) were higher compared to those of the IDF (OR = 1.64; 95 % CI: 1.39–1.94) and the ATP III (OR = 1.48; 95 % CI: 1.32–1.67) (Table 2).

**Table 2:** Summary of odds ratio estimates [95 % CIs] for cross-sectional studies focused on the association between depression and risk of MetS by gender, study population, continent, depression scales, MTs Scales, and Age

Subgroup	Number of studies	Summary OR (95 % CI)	Between studies			Between subgroups	
			I^2^	P _heterogeneity_	Q	Q	P _heterogeneity_
Gender Female Male Both	5 5 22	1.35 (0.90– 2.01) 1.52 (1.13– 2.05) 1.49 (1.31–1.69)	70.37 % 0.00 % 59.94 %	0.01 0.99 0.01	14.48 0.27 52.42	0.26	0.88
Continents America Asia & Australia Europe	6 16 9	1.45 (0.94–2.25) 1.37 (1.26–1.49) 1.71 (1.47–1.99)	79.28 % 13.03 % 21.69 %	0.01 0.30 0.26	28.96 20.70 7.66	6.38	0.04
Depression Scales BDI CES-D Interview & DSM PHQ-9 Others	5 2 11 5 8	1.52 (1.19–1.94) 1.20 (0.70–2.05) 1.70 (1.37–2.12) 1.49 (1.34–1.66) 1.36 (1.09–1.69)	0.00 % 76.72 % 50.54 % 67.90 % 0.00 %	0.88 0.01 0.03 < 0.001 0.44	0.25 8.59 20.22 28.03 4.77	2.76	0.60
MTs Scales ATP III IDF WHO	27 4 2	1.48 (1.32–1.67) 1.64 (1.39–1.94) 2.00 (0.53–7.52)	50.56 % 0.00 % 71.85 %	< 0.001 0.68 0.06	52.69 1.51 3.55	1.09	0.58
Study Population General Population Patients Other	27 4 2	1.46 (1.33–1.60) 1.95 (1.38–2.74) 0.73 (0.18–2.88)	35.25 % 58.25 % 89.16 %	0.04 0.07 < 0.001	40.15 7.19 9.22	3.54	0.17
Age ≤ 50 Year > 50 Year	5 7	1.66 (1.41–1.94) 1.39 (1.00–1.95)	42.62 % 50.97 %	0.14 0.06	6.97 12.24	0.82	0.36

Abbreviations: *BDI-I* Beck Depression Inventory; *CES-D* Center for Epidemiologic Survey-Depression; *DSM* Diagnostic and Statistical Manual of Mental Disorders; *IDF* International Diabetes Federation; *MHI-5* 5-item Mental Health Inventory; *NCEP-ATP III* National Cholesterol Education Program-Adult Treatment Panel III; *PHQ* Patient Health Questionnaire; *WHO* World Health Organization

### Meta‐analysis of cohort studies

#### Depression and risk of metabolic syndrome

**Fig. 4:**
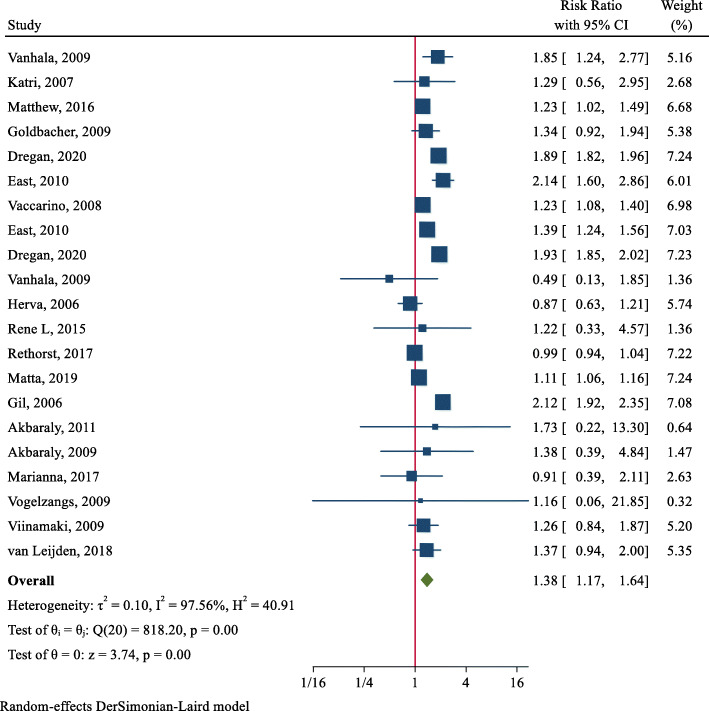
Forest plot for the pooled Risk Ratio of the association between depression and MetS in the general population

Analysis of cohort studies showed that the pooled Risk Ratio of MetS in patients with depression was 1.38 (95 % CI: 1.17–1.64). Heterogeneity was found to be 97.56 %, and the Cochran’s Q test led to a statistically significant result (Q = 818.20, DoF = 20, *p* < 0.001) (Fig. 4).

#### Subgroup analysis

Results of subgroup analysis by continent showed that pooled ORs were higher in the studies conducted in America (OR = 1.46; 95 % CI: 1.16–1.84) compared to those conducted in Europe (OR = 1.28; 95 % CI: 0.95–1.73). Heterogeneity was significant for the cohort studies conducted in America (I^2^ = 98.51 %) and Europe (I^2^ = 92.41 %) (p < 0.001). Results by gender showed that depressed men were more likely to develop MetS compared to depressed women [OR = 1.55; 95 % CI: 1.23–1.94) vs. (OR = 1.54; 95 % CI: 1.11–2.14)]. Additionally, participants under 50 years of age were more likely to develop MetS (OR = 1.30; 95 % CI: 1.13–149) compared to those over 50 years. Results of subgroup analysis also showed that pooled ORs were higher in the studies assessing depression using the BDI (OR = 1.50; 95 % CI: 1.02–2.20) compared to those assessing this variable using other scales. Moreover, pooled ORs of the ATP III criteria (OR = 1.41; 95 % CI: 1.18–1.69) for diagnosis of MetS were higher compared to those of the IDF criteria (OR = 1.13; 95 % CI: 1.01–1.27) (Table 3).

**Table 3:** Summary of relative risk estimates (RR) (95 % CIs) for cohort studies that assess the association between depression and risk of MetS by gender, study population, continent, depression scales, MetS scales, and Age.

Subgroup	Number of studies	Summary Relative Risk (95 % CI)	Between studies			Between subgroups	
			I^2^	P _heterogeneity_	Q	Q	P _heterogeneity_
Gender Female Male Both	7 3 11	1.55 (1.23–1.94) 1.54 (1.11– 2.14) 1.23 (0.99–1.53)	89.04 % 93.61 % 94.47 %	< 0.001 < 0.001 < 0.001	59.64 31.28 89.61	2.40	0.30
Continents America Europe	9 12	1.46 (1.16–1.84) 1.28 (0.95–1.73)	98.51 % 92.41 %	< 0.001 < 0.001	99.79 95.84	0.45	0.50
Depression Scales BDI CES-D Interview& DM Other	5 8 5 3	1.50 (1.02–2.20) 1.34 (1.11–1.61) 1.45 (1.05–2.00) 1.10 (0.77 – 1.56)	91.39 % 77.66 % 99.21 % 38.96 %	< 0.001 < 0.001 < 0.001 0.19	46.48 31.34 98.27 3.28	1.81	0.61
MetS Scales ATP III IDF	19 2	1.41 (1.18–1.69) 1.13 (1.01–1.27)	96.94 % 14.80 %	< 0.001 0.28	588.0 1.17	4.14	0.04
Study Population General Patients Other	19 1 1	1.46 (1.33–1.60) - -	97.78 % - -	< 0.001 - -	810.7 - -	1.32	0.52
Age ≤ 50 Year > 50 Year	11 2	1.30 (1.13–1.49) 1.21 (1.13–1.44)	84.26 % 0.00 %	< 0.001 0.49	63.54 0.47	0.34	0.59

**Fig. 5:**
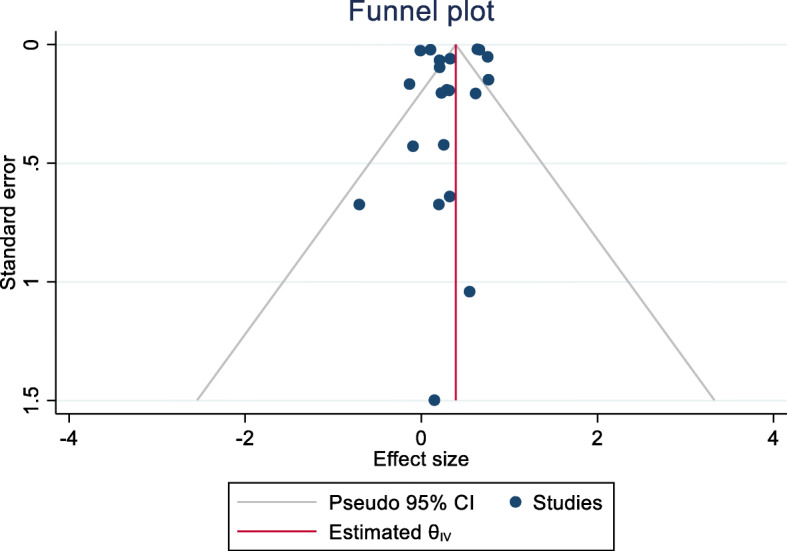
Funnel plot for testing for publication bias in the pooled estimate of the effect of depression on the risk of MetS in the general population

Also, meta-regression analysis indicated that the log β (risk ratio) of MetS in patients with depression is not associated with age (β: -0.011, SE: 0.024, *p* = 0.637, 95 % CI: − 0.059, − 0.036). Additionally, the funnel plot and the results of Egger test (Coefficient: -0.44, SE: 0.449, *p* = 0.331) indicated no publication bias in the studies (*p* = 0.437) (Fig. 5).

## Discussion

Our meta-analysis of 31 cross-sectional and 18 cohort studies indicted a significant relationship between depression and MetS. The results of a previous meta-analysis in which 16 articles were analyzed showed that there was a relationship between metabolic syndrome and depression. The present meta-analyses showed that depressed patients were more likely to have MetS compared to non-depressed patients [58–60]. In the previous meta-analysis [58], 16 articles were reviewed, but the articles analyzed in this study had increased to 49 cases. The increase in the number of articles in this field indicates the interest of researchers and it seemed necessary to re-examine the controversial relationship between these variables.

Although the mechanism of this association is unknown, some hypotheses have been suggested. Depression can lead to MetS through different mechanisms. Firstly, depressed patients tend to have adverse health behaviors, such as alcohol consumption, cigarette smoking, poor diet, and may have a sedentary lifestyle that all can have a role that leads to MetS [30, 58]. Secondly, depressed patients are less likely to follow their treatment regimen [61]. Results of a meta-analysis by DiMatteo et al. (2000) showed that depressed patients were twice as less likely to adhere to treatment than non-depressed individuals [62]. Thirdly, antidepressants may lead to MetS through increasing the risk of abdominal obesity, high blood pressure, and increased triglycerides levels [63]. Akbaraly indicated that there is a mutual association between depression and MetS; he calls this association a “two-way street” [54]. Theoretically, depression can activate the hypothalamic–pituitary–adrenal axis and lead to accumulation of visceral fat through increasing the secretion of corticotrophin-releasing hormone, adrenocorticotropic hormone, and cortisol [50].

Moreover, results of cross-sectional studies indicated that the pooled ORs of MetS were higher in depressed patients than in the general population, and higher in America than in Asia. Also, in the included cohort studies, the risk of MetS in depressed patients with underlying conditions was higher than in the General population and higher in America compared to Europe. This finding can be attributed to the type of study methodology and the characteristics of the studied samples. Various studies have shown an association between depression and diet [64–66]. Le Port et al. indicated that fruits and fish diet was related to lower risk of depression symptoms [67]. Huang et al. (2019) have also shown that a healthy diet such as a Mediterranean diet and certain foods such as fish, fresh vegetables, and fruits can reduce depression [68]. Also, Allison stated that in populations with different sociocultural backgrounds, there are different risk factors as a result of different genetic and socioeconomic factors [69].

Results of the included cross-sectional studies by gender indicated that pooled ORs were higher in studies conducted in men compared to those conducted in women or mixed group of men and women. Results of cohort studies showed that the risk of MetS was almost the same in men and women. Results of some studies were influenced by gender; for example, in some studies, the association between depression and MetS was only significant in men [13, 56], while in some others, this association was only significant in women [30, 37, 55, 70]. This finding can be explained by the fact that compared to women, men are more likely to have an unhealthy lifestyle, such as drinking alcohol or eating fast food, tend to pay less attention to their appearance and weight, and may be less willing to go to the doctor when experiencing physical problems [71, 72].

The results of cross-sectional studies showed that pooled ORs of MetS in depressed patients were higher in studies assessing depression using interviews and assessing MetS using the WHO’s criteria compared to studies assessing these two conditions using other tools or criteria. The cohort studies that used the BDI and the ATP III to screen for depression and MetS reported a higher risk of MetS in depressed patients compared to those that used other tools or criteria. Only two cross-sectional studies used the WHO criteria; this may have influenced the generalizability of their results. Most of the cohort studies used the ATP III criteria to diagnose MetS which is more common than the other criteria. According the regression analysis of cross-sectional and cohort studies, the risk of MetS in depressed patients decreased with age; further studies are needed to explain this finding.

Some strengths of the present study includes the focus on a new topic, extra study details, large number of studies, and large number of patients included in the meta-analysis.

## Conclusions

The results of the present systematic review and meta-analysis indicated a relationship between depression and metabolic syndrome (MetS). Understanding this association is important because a history of depression, which predicts the risk of cardiovascular disease in the future, that can worsen underlying health conditions and may increase mortality rates.

## Data Availability

The datasets used and/or analyzed during the current study are available from the corresponding author on reasonable request.
